# Effects of Kamishoyosan, a Traditional Japanese Kampo Medicine, on Pain Conditions in Patients with Intractable Persistent Dentoalveolar Pain Disorder

**DOI:** 10.1155/2015/750345

**Published:** 2015-10-01

**Authors:** Young-Chang P. Arai, Izumi Makino, Shuichi Aono, Hiromichi Yasui, Hideya Isai, Makoto Nishihara, Noboru Hatakeyama, Takashi Kawai, Tatsunori Ikemoto, Shinsuke Inoue, Takahiro Ushida

**Affiliations:** Multidisciplinary Pain Center, School of Medicine, Aichi Medical University, Aichi 480-1195, Japan

## Abstract

There are patients who suffer from persistent dentoalveolar pain disorder (PDAP) which is a pain of the teeth, either dentoalveolar pain or nonodontogenic toothache, and its cause has not yet been identified. An effective intervention for PDAP has not yet been established. Interventions for patients with PDAP are generally pharmacological treatments such as antidepressants, anticonvulsants, and pregabalin. However, these medicines are not always effective for patients. The pain disorder in the orofacial region including temporomandibular disorder (TMD) and PDAP was effectively treated with our original exercise therapy. However, we did observe some intractable cases of PDAP even when our original exercise therapy was used. This paper presents our findings in which Kamishoyosan improved the pain intensity in 14 out of 15 PDAP patients refractory to our original exercise therapy.

## 1. Introduction

Patients suffering from tooth pain, either dentoalveolar pain or nonodontogenic toothache, of which the cause has yet to be identified, continue to seek pain treatment from several doctors, not only dentists but also several kinds of medical practitioners.

Recently the name of persistent dentoalveolar pain disorder (PDAP) has been used to describe this kind of pain. PDAP is described as a pain that persists in the dentoalveolar region without any evidence of local disease [1–4]. Much attention has been paid to PDAP among dentists who have knowledge of orofacial pain, but PDAP is not widely known because of the lack of diagnostic criteria and pathology. Previous reports suggested that the pain disorder in the orofacial region including PDAP could be associated with psychological problems [5, 6]. Moreover, PDAP is supposed to be partly somatoform pain and partly neuropathic pain. Thus, typical interventions for patients with PDAP especially through pharmacological treatment are antidepressants [6], anticonvulsants [7], and pregabalin [8]. However, these medicines are not always effective for patients and some patients experience severe side effects. We have effectively treated the pain disorder in the orofacial region including temporomandibular disorder (TMD) and PDAP by using our original exercise therapy for the improvement of jaw movement and psychological intervention (PI) to reduce parafunctional activities (PAs) in our daily clinical practice [9]. Patients with not only TMD but also PDAP display oral PAs. PAs indicate oral habitual movements (such as tongue habit, masticating on one side, lip/cheek biting and swallowing) and bruxism (clenching, grinding, and tapping of teeth). However, we experience intractable cases in a third of patients with PDAP even when our original exercise therapy is used.

Kampo, traditional Japanese herbal medicine based on traditional Chinese herbal medicine, has been used to treat chronic pain in Japan [10]. Kamishoyosan is a Kampo formula used to effectively treat psychological symptoms such as anxiety, irritability, and depression [10–12]. Therefore, we considered that Kamishoyosan might relieve their psychological problems and thereby be effective for the PDAP patients with intractable pain after our original exercise therapy. We report here that Kamishoyosan improved the pain intensity in 14 out of 15 PDAP patients refractory to our original exercise therapy.

## 2. Case Series

After obtaining approval from the Ethics Committee of Aichi Medical University for the review of the use and effect of Kampo medicine (reference number: 13-097), retrospective analysis from January 2012 to April 2013 was performed on 15 patients who visited the Multidisciplinary Pain Center (Pain Center) of Aichi Medical University. All patients were referred from other hospitals to the Pain Center because of PDAP. Treatment protocols used in the present report were based on institutional policy and clinical guidelines approved by the Ethics Committee of Aichi Medical University. The characteristics of the patients are described in Table 1. Fifteen patients were female, and their ages ranged from 35 to 76 years.

After obtaining approval from the Ethics Committee of Aichi Medical University, we routinely explain to all patients that we record and store demographics, symptoms, course of pain, and medical record in all patients for possible future use in our research and then we obtain written informed consent at their initial visit to the Pain Center. We diagnosed each patient's condition as PDAP based on their medical histories and radiographic examinations. The intensity of pain was rated by the patients using a numerical rating scale (NRS) where 0 indicated “no pain” and 10 “the greatest pain possible.” The jaw movement test was evaluated using a cotton roll [9]. We administered our unique exercise therapy to the patients under the supervision of a dentist (MAKINO) at our clinic for approximately 20 minutes during each visit. Patients were ordered to continue the exercises at home. All patients visited our Pain Center on a monthly basis.

## 3. Treatment

*(1) Explanation*. Firstly, we confirmed that there was no clinical and radiographic evidence of relevant pathology in their pain and explained this to the patients [9].

**Figure 1:**
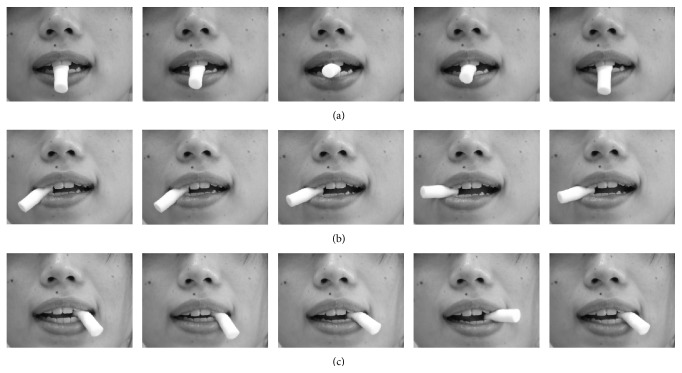
(a) Protrusion-retrusion jaw movements while biting on a cotton roll with the front teeth. (b) Right jaw movement while biting on a cotton roll with the right canine. (c) Left jaw movement while biting on a cotton roll with the left canine.

*(2) Exercise Therapy. *Patients were instructed to perform the jaw movement exercise at home. The exercise consists of ten sets of protrusion-retrusion (anterior-posterior) jaw movements, a lateral jaw movement with the right side and then a lateral jaw movement on the left side (Figure 1) [9]. The exercises were performed once a day. It was important for the patients to concentrate on the jaw exercise while watching their jaws move in a mirror and to move the jaw slowly.

**Figure 2:**
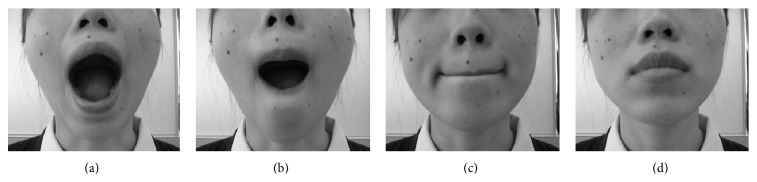
Relaxation training. (a) Take a deep breath with the mouth wide open. (b) Roll the lips back inside the mouth. (c) Close the mouth until the lips touch. (d) Relax the masticatory muscles, tongue, and jaw without letting the teeth touch.

*(3) Psychological Intervention*. We let patients understand that it was important to recognize both PAs and frequency of PAs during the daytime. Our only instructions were that when they felt pain, they had to recognize what kind of PAs they were doing and how frequently they were doing them. In addition, the patients were trained to relax their tongue, their masticatory muscles, and their jaw (Figure 2) [9]. They were instructed to perform this form of relaxation when they noticed their pain. Moreover, they were instructed to try and feel pain relief when they avoided their PAs and relaxed. In addition, they were told to take notice of what kind of PAs they were doing if they felt pain.

The fifteen patients had not experienced any improvement in the intensity of their pain two months after commencing our original exercise therapy and PI (Table 1). Thus, we proposed administering them a course of Kampo medicine, a traditional Japanese herbal medicine. We explained to the patients that we recorded and stored their demographics, symptoms, course of pain, and medical record for future use in our review again and then we obtained written informed consent. Patients agreed to take Kamishoyosan. They took 2.5 g of Kamishoyosan (TJ-24, Tsumura & Co., Tokyo, Japan) three times a day for two weeks, while continuing the exercise therapy and psychological intervention to reduce parafunctional activities. The individual changes of NRS are described in Table 2. NRSs of the baseline ranged from 3 to 10 (Median, 7). Eleven patients displayed jaw movement abnormalities while four patients had normal functions using our original jaw moving test. Twelve patients displayed PA habits. The NRS significantly decreased two weeks later (Median, 0; range 0–6; Wilcoxon matched pairs test,* P* = 0.001). Intensity of pain improved in 14 out of the 15 patients. Although several patients reported that the pain did not completely disappear, if they felt any pain it was not at a level which caused them concern. Eight patients improved but three patients did not improve in jaw movement. No patient experienced any adverse effects during the medication. Although ten patients experienced recurrences of pain in about a year, Kamishoyosan provided an improvement of PDAP at each recurrence.

## 4. A Particular Case

A 48-year-old female had been suffering from severe spontaneous pain (NRS, 10) and sensitivity to cold in the upper right maxillary first molar for 2 months. Firstly, we confirmed that there was no clinical and radiographic evidence of relevant pathology in her pain and explained this to her. She displayed jaw movement abnormality. She had not experienced any improvement in the intensity of her pain (NRS, 10) two months after commencing our original exercise therapy and PI. She took 2.5 g of Kamishoyosan three times a day, while continuing the exercise therapy and PI to reduce parafunctional activities. The NRS dramatically decreased to 4 four days later and was 0 at day 11. Also, she improved in jaw movement. Although she experienced two recurrences of pain in a year, Kamishoyosan provided a quick improvement of PDAP at each recurrence.

## 5. Discussion

Many studies have reported the association between PDAP and TMD [6, 13]. Patients with the pain disorder in the orofacial region including TMD and PDAP have PAs [9]. PAs indicate oral habitual movements (such as tongue habit, masticating on one side, lip/cheek biting, and swallowing) and bruxism (clenching, grinding, and tapping of teeth). Thus, we focused our attention on jaw movement and PAs which are relevant factors in chronic pain of the craniocervical region. We found that many patients showed abnormalities in our jaw moving test and recognized PAs while experiencing pain. Previous reports suggested that PAs are associated with emotional stress induced by social factors [14] and that PAs are recognized as a valid systemic prophylactic reaction in all stress-related diseases [13, 15]. Moreover, many studies reported that chronic pain disorders including PDAP have been linked to psychological factors [5, 13].

Although therapeutic algorithms for PDAP [2] include pharmacological treatments which are tricyclic antidepressants [6], serotonin, and norepinephrine reuptake inhibitors [16], anticonvulsants [7] or pregabalin [8], sometimes these treatments cause serious side effects and are not highly effective with treating PDAP. In fact, since a lot of patients do not experience sound pain relief by pharmacological treatment in our clinical practice, firstly we have been trying to treat them by using our original exercise therapy and PI. In two thirds of them, the pain intensity decreases by more than two of NRS in two weeks and further decreases in a few months, and they report that the pain does not completely disappear (NRS, 0–3), but if they feel any pain it is not at a level which causes them concern. However, in the present report, the fifteen patients had not experienced any improvement in the intensity of their pain two months after commencing our original exercise therapy and PI.

Kampo, or traditional Japanese herbal medicine, has been used for the treatment of not only chronic pain but also many other diseases in Japan. Kamishoyosan is a Kampo formulation used to effectively treat psychological symptoms such as anxiety, irritability, and depression [10–12]. In the present report, although the fifteen patients had not experienced any improvement in the intensity of their pain two months after commencing our original exercise therapy and PI, the simultaneous use of a Kampo formulation with education and psychological intervention provided an improvement in patients with PDAP. We thus postulate that the addition of the Kampo formulation, Kamishoyosan, to our exercise therapy could effectively improve PDAP.

There are several limitations in the present report. The present report is a small case series and not a nonrandomized control analysis of Kampo treatment. Another limitation is that the male-female ratio of PMDA is 1 : 9 in our clinical practice, so all 15 patients were female in the present report. Although Kampo medicines must basically be prescribed for patients based on patient-centered Kampo diagnosis, we did not see if the 15 patients matched with Kamishoyosan pattern. Thus, we need a prospective and comparative study that might be more appropriate to support a predictive, preventive, and personalized value of Kampo treatment.

## 6. Conclusion

We treated 15 PDAP patients who were refractory to our unique exercise therapy to improve jaw movement and PI to reduce PAs through administration of the Kampo formulation, Kamishoyosan. Fourteen patients experienced a decrease in pain intensity after intervention. This finding suggests that the combination of Kampo and our exercise therapy was effective in treating PDAP.

## Figures and Tables

**Table 1:** Characteristics in 15 patients. Parafunctional activities comprise oral habitual movements (such as tongue habit, masticating on one side, lip/cheek biting, and swallowing) and bruxism (clenching, grinding, and tapping of teeth).

	Age (years)	Sex	Duration (month)	Pain	Pharmacological treatment	Parafunctional activities
1	35	F	2	Tooth pain	NSAIDs	−
2	37	F	6	Tooth pain	NSAIDs and muscle relaxants	+
3	40	F	1	Tooth pain	NSAIDs	−
4	46	F	60	Tooth pain	NSAIDs	+
5	48	F	2	Tooth pain	NSAIDs	+
6	52	F	180	Tooth pain	NSAIDs and antidepressants	+
7	55	F	6	Tooth pain	NSAIDs	+
8	56	F	1	Tooth pain	NSAIDs	+
9	56	F	1	Tooth pain	NSAIDs	+
10	64	F	48	Tooth pain	—	+
11	64	F	12	Tongue and alveolar bone	Muscle relaxants and antidepressants	+
12	65	F	48	Tooth pain	—	+
13	68	F	180	Tooth pain	Muscle relaxants and antidepressants	−
14	70	F	12	Tooth pain	Antidepressants	+
15	76	F	192	Tongue and alveolar bone	Muscle relaxants and antidepressants	+

**Table 2:** Results of patients' individual pain scores assessed by numerical rating scales (NRS), jaw movement tests, and recurrence of pain.

	NRS pre	NRS day 14	Jaw movement test pre	Jaw movement test day 14	Recurrence
1	6	0	Abnormal	Normal	+
2	8	0	Normal	Normal	+
3	7	1	Abnormal	Normal	+
4	5	0	Normal	Normal	+
5	10	0	Abnormal	Normal	+
6	7	5	Abnormal	Abnormal	+
7	10	3	Abnormal	Normal	+
8	8	1	Normal	Normal	−
9	5	0	Abnormal	Normal	−
10	5	0	Abnormal	Normal	−
11	9	5	Abnormal	Normal	+
12	8	0	Normal	Normal	−
13	6	6	Abnormal	Abnormal	−
14	5	0	Abnormal	Normal	+
15	3	0	Abnormal	Abnormal	+
